# Safe Management of Adverse Effects Associated with Prescription Opioids in the Palliative Care Population: A Narrative Review

**DOI:** 10.3390/jcm13102746

**Published:** 2024-05-07

**Authors:** Amanda Zimmerman, Adam Laitman

**Affiliations:** 1 West Forsyth Pain Management, Winston Salem, NC 27103, USA; 2 Salix Pharmaceuticals, Bridgewater, NJ 08807, USA; adam.laitman@salix.com

**Keywords:** analgesics, opioid, constipation, palliative care, safety

## Abstract

In the palliative care population, prescription opioids are often considered viable pain relief options. However, in this complex patient population, the adverse effects of opioid medications should be identified and managed without delay. Common adverse effects can include constipation, nausea, somnolence, dizziness, vomiting, and pruritus. Less common adverse effects can include potentially lethal respiratory depression and cardiovascular effects. Critical aspects of safe opioid prescribing are recognition of side effects and knowledge of effective management strategies; prompt management is necessary for uninterrupted pain relief. Most complications are managed with general approaches such as dose reduction, opioid rotation, alternate routes of administration, and symptomatic management. The only opioid-induced complication for which US Food and Drug Administration-approved treatments currently exist is constipation. Treating laxative-refractory opioid-induced constipation (OIC) with peripherally acting mu-opioid receptor antagonists (PAMORAs), which block gastrointestinal opioid receptors, can restore gastrointestinal motility and fluid secretion. This narrative review discusses key complications of prescription opioid treatment and their management in the palliative care setting.

## 1. Introduction

In certain patient populations, prescription opioid analgesics are considered viable options for pain relief [1]. Those populations include individuals with cancer [2], those receiving palliative care [3,4], those with other advanced illnesses [1,3], and those with cardiovascular, renal, hepatic, or gastrointestinal comorbidities and chronic pain who may not be candidates for treatment with nonopioid pain medications due to their associated risks [1,5]. This narrative review will discuss key complications of prescription opioid treatment and how they may be managed in the palliative care setting.

Palliative care is defined by the World Health Organization as “an approach that improves the quality of life of patients (adults and children) and their families who are facing problems associated with life-threatening illness” [6]. The goal of palliative care is to relieve suffering from the symptoms and side effects of a disease and its treatment, and palliative care can be given in conjunction with active treatment or when active treatment is no longer used [7]. Thus, the palliative care population is quite diverse, as it contains individuals of a broad age range with a wide variety of diseases, complications, and comorbidities. A systematic review found that the estimated prevalence of chronic, nonmalignant pain among patients receiving various types of palliative care ranged from 14% to 34% [8], and in one study, ~22% of individuals receiving palliative home care reported severe daily pain in their last month of life [9]. 

To make appropriate decisions when designing pain management plans for individuals receiving palliative care, it is important to consider the risk/benefit ratio of prescription opioid medications and the management of their potential adverse effects. Of the many adverse effects associated with the use of prescription opioids, the most common are constipation, nausea, somnolence, dizziness, vomiting, and pruritus, and less common but potentially lethal adverse effects include respiratory depression and cardiovascular complications [1]. Some opioid-induced adverse effects are related to genetic factors, such as polymorphisms in cytochrome P450 2D6 (CYP2D6) and other drug-metabolizing enzymes that affect the metabolism and disposition of specific agents [10]. 

Opioids bind to four main classes of receptors: mu, kappa, delta, and the non-classical nociception opioid receptor [11]. Analgesia is mediated primarily through the mu receptors present in the central nervous system (CNS); however, all opioid receptors influence analgesia to some extent. The stimulation of peripheral mu opioid receptors leads to adverse effects, such as constipation, which results from stimulation of gastrointestinal mu opioid receptors, while the adverse effects of nausea, vomiting, respiratory depression, and tolerance are mediated via mu receptors in the CNS [11]. Stimulation of CNS delta opioid receptors produces antidepressant and anxiolytic effects [12], as well as contributing to the adverse effects of nausea and vomiting [11]. In the gastrointestinal system, delta opioid receptors are involved in gastrointestinal motility. Kappa receptors mediate neuroendocrine functions but are also associated with the adverse effects of dysphoria and psychotomimesis [11].

Prompt management of opioid-induced adverse effects is necessary for optimal pain relief and improved quality of life. Results of a cohort study of patients with moderate to severe cancer pain who initiated treatment with strong opioids showed that those who experienced ≥ 4 opioid-induced adverse effects had worse overall clinical symptoms and significantly less pain relief compared with those who experienced few or no opioid-induced adverse effects [13]. Inadequate pain relief may have additional physiological and psychological effects in patients receiving palliative care, as has been found in other patient populations; for example, in patients with breast cancer with inadequately treated postsurgical pain, increased heart rate and blood pressure, impaired immune function, muscle weakness, thromboembolism, and anxiety were observed [14].

## 2. Opioid Availability and Eligibility in Palliative Care

Recently published expert consensus recommendations for safe opioid use in the palliative care population point out that because palliative care is now initiated much earlier than the end of life for some patients with advanced cancer and other life-threatening diseases, patients receiving long-term, high-dose opioid therapy may be at an increased risk for opioid-related harms [4]. Recommendations to promote opioid safety address the identification, prevention, and management of aberrant medication-taking behaviors, opioid use disorder, and opioid overdose, and include strong support for assessments of alcoholism, nonmedical drug use, injection drug use, post-traumatic stress, sexual abuse history, and criminal records related to substance misuse to identify those at high risk of aberrant medication-taking behaviors and assessments of alcohol and benzodiazepine use, history of opioid overdose, history or current substance use disorder, and receipt of opioid prescriptions from ≥2 physicians to identify those at high risk of opioid overdose [4].

The National Comprehensive Cancer Network (NCCN) Guidelines for Palliative Care note that the risk/benefit of treatment options should be reviewed with the patient, the family, and the caregiver [3], and the NCCN Guidelines for Adult Cancer Pain provide recommendations for opioid prescribing in terms of potential risk factors for opioid misuse [2]. In addition, substance misuse experts have recommended implementing universal precautions, such as (1) conducting a thorough assessment of a patient’s past and present use of legal and illicit substances and cannabis; (2) establishing agreements regarding the treatment plan, use of one pharmacy and one prescriber, and safe storage of medication; and (3) monitoring adherence via pill counts and toxicology screening, to avoid prescribing opioids to a patient at risk of opioid misuse [15]. Although not developed as a guide for opioid prescribing for patients receiving palliative care, the CDC Clinical Practice Guideline for Prescribing Opioids for Pain contains suggestions for determining patient eligibility for opioid treatment (e.g., establishing medical necessity based on assessments of pain, function, and risk) [1]. The Opioid Risk Tool developed by the National Institute on Drug Abuse (available at: https://nida.nih.gov/sites/default/files/opioidrisktool.pdf (accessed on 25 April 2024)) is a validated, self-administered rapid screening tool that is widely used to assess the risk of opioid misuse in patients with chronic pain.

## 3. Management of Opioid-Related Adverse Effects

Ongoing assessment of risk, as well as recognition and management of side effects, are critical aspects of safe and appropriate opioid prescribing. Prior to the initiation of opioid therapy, education of the patient and caregiver regarding the beneficial and adverse effects of opioid therapy should be the focus. Reassessment of risk and adverse effects should be carried out at every visit [1,2,3,16]. It is important to note that in the palliative care population, differences in drug safety will be observed among the numerous disease states represented due to the expected diversity of drug effects on the body. 

General strategies for avoiding or reducing the incidence and severity of side effects include dose reduction, opioid rotation, changing the route of administration, and symptomatic management, although not all strategies apply to all side effects [2,17,18]. Opioid-related adverse effects are discussed below, and those for which frequencies have been reported are shown in Table 1. Both the fragile nature of the palliative care population and the common use of polypharmacy in these patients must be considered when determining management strategies for opioid-induced adverse effects.

### 3.1. Cognitive Effects

The central nervous system effects of opioids on cognition and delirium are most common at treatment initiation, typically occurring with dose escalation. In this patient population, opioid-induced cognitive effects may be difficult to distinguish from the impairment/delirium that is common with terminal illness or from the effects of comorbid conditions (e.g., renal impairment, dehydration, use of other drugs). Potential management approaches include a reduction in the opioid dose and the addition of adjuvant analgesics, as well as pharmacologic treatment (e.g., typical and atypical antipsychotics) to address these symptoms [2]. Consultation with a neuropsychiatrist or behavioral health professional should be considered.

### 3.2. Endocrine Effects

Opioids suppress the hypothalamic–pituitary–adrenal axis to reduce gonadal function [24]. The direct action of opioids on the hypothalamus reduces the release of gonadotropin-releasing hormone and thereby adversely affects levels of luteinizing hormone and, subsequently, testosterone synthesis and secretion [24]. Symptoms include infertility, decreased sexual function, loss of muscle mass, and anxiety and/or depression. These changes develop within weeks to years. Thus, endocrine function should be monitored in the setting of chronic prescription opioid therapy. Management options include tapering and withdrawing opioid treatment; the benefit of switching opioids is not known. Testosterone replacement therapy may also be used [16,24].

### 3.3. Gastrointestinal Effects

#### 3.3.1. Constipation

The mechanism underlying opioid-induced constipation (OIC) involves the stimulation of gastrointestinal mu, kappa, and delta opioid receptors, which leads to decreases in motility and fluid secretion and the development of constipation [25]. OIC is one of the most common adverse effects of opioid treatment, and unlike other opioid side effects, OIC is not dose-dependent; thus, it is not alleviated by dose reduction, and patients do not develop a tolerance to OIC [16]. Serious outcomes of untreated OIC include fecal impaction and stercoral perforation [26,27]. In the palliative care population, OIC may be aggravated by polypharmacy, dehydration, and electrolyte disturbances.

Guidelines for the treatment of OIC recommend laxatives as a first-line option [2,28]. Stimulant laxatives, such as senna, are recommended for prophylaxis, and combinations of laxatives that act by different mechanisms are recommended for persistent constipation [29]. Laxatives act by allowing water and lipid penetration of the stool (stool softening laxatives), by promoting colonic motility and reducing colonic absorption of water (stimulant laxatives), and by increasing intestinal fluid secretion (chloride channel activators) [28]. Other interventions for OIC include manual disimpaction, dietary changes, relaxation techniques, and the use of suppositories or enemas [30].

In contrast to these general approaches, which may help to alleviate the end result of gastrointestinal mu-opioid receptor stimulation, peripherally acting mu-opioid receptor antagonists (PAMORAs) were designed specifically to target the molecular mechanism underlying the development of constipation caused by an opioid. The mechanism of action of PAMORAs is to inhibit opioid binding to gastrointestinal mu-opioid receptors, which enables the enteric nervous system to function to maintain gastrointestinal motility and fluid secretion during opioid treatment [28]. Current guidelines recommend PAMORAs for the treatment of laxative-refractory OIC [2,28]. To date, PAMORAs are the only medications designed specifically to treat OIC, and constipation is the only opioid-induced complication for which US Food and Drug Administration (FDA)-approved prescription treatments are indicated. Three PAMORAs currently approved by the US FDA are summarized in Table 2. 

#### 3.3.2. Nausea/Vomiting

The causes of opioid-induced nausea and vomiting are multifactorial and include opioid stimulation of receptors in the gastrointestinal tract, the vestibular apparatus, the cerebral cortex, and the chemoreceptor trigger zone for vomiting, each of which provides input to the vomiting center in the medulla oblongata [16,34]. Bowel obstruction may also lead to nausea and should be assessed in patients with nausea [29]. Treatment with antiemetic agents (e.g., prochlorperazine, metoclopramide) is recommended in treatment guidelines and is often effective [2,16]. However, caution must be exercised when prescribing promethazine, given its potentiation of the sedating effects of opioid medications (see below) and its potential for misuse when administered concomitantly with opioids [35]. Alternative agents recommended for the treatment of nausea may include ondansetron, olanzapine, scopolamine, dexamethasone, and mirtazapine, depending on patient-specific factors [29]. Nausea and vomiting appear early after treatment initiation, and tolerance may develop over time [2,16].

### 3.4. Other Adverse Effects

#### 3.4.1. Sedation

Sedation is a common, well-known side effect of opioid treatment. Mediated by the mu opioid receptor [36], sedation typically occurs at therapy initiation or with increases in dose [19]. Opioid treatment, in conjunction with other CNS depressants, can exacerbate sedative effects. Special care should be taken to avoid concomitant use of other CNS depressants with opioid therapy, with special consideration for benzodiazepine therapy in addition to the opioid. Patients may become tolerant of the sedative effects of their opioid regimen over time. Opioid rotation, more frequent administration of lower opioid doses, and the use of caffeine, methylphenidate, dextroamphetamine, modafinil, or armodafinil may be effective in ameliorating opioid-induced sedation [2,19].

#### 3.4.2. Tolerance/Loss of Analgesia

Tolerance to the analgesic effects of opioids is common and leads to constantly increasing dose requirements and decreasing drug effectiveness over time. N-methyl-D-aspartate (NMDA) receptors, along with opioid receptor internalization and degradation, decreased opioid receptor numbers, and changes in neural pain modulatory circuits with long-term use [18,20,37]. Opioid rotation may be a good option to address this effect due to a lack of complete cross-tolerance across opioids [18]. Tolerance may also be addressed by using non-opioid analgesics and adjuvants, adding them to opioid treatment, and changing the opioid dosing schedule [29].

#### 3.4.3. Myoclonus

Myoclonus is an occasional side effect of opioid therapy that involves uncontrolled movement of the limbs, may be dose-dependent, and may occur more frequently with concomitant use of neuroleptics, antidepressants, and antiemetics. The mechanism underlying this effect is not well understood but is thought to involve loss of glycine inhibition, NMDA receptor activation, and dopamine. Potential management options include opioid rotation and the administration of benzodiazepines and gabapentinoids [16]. However, the prescribing information for both benzodiazepines and opioids contains a black box warning stating that concomitant use of these agents increases the risk of fatal overdose [38]; thus, they should be co-prescribed with caution.

#### 3.4.4. Pruritus

Opioid-induced pruritus is considered to be mediated by the activation of mu receptors, but its mechanism is not well understood [16]. It is thought that opioid-induced histamine release from mast cells may be involved in some instances [39]. Pruritus is commonly observed with epidural or intrathecal opioid preparations. Potential treatments include antihistamines, continuous infusions of naloxone, opioid rotation, and opioid discontinuation [2,16].

#### 3.4.5. Psychomotor Effects

Psychomotor performance may decrease with the initiation of opioid therapy. Performance scores may be improved with psychostimulants, such as methylphenidate. Psychomotor effects decrease in incidence over time with the development of tolerance [18]. Because these effects may interfere with driving, patients should be evaluated for impairment [2]. Whether patients receiving opioid therapy should be allowed to drive is a subject of controversy, as those on stable regimens may have no significant cognitive or psychomotor impairments [18]. Monitoring of these functions is required for prudent decision-making [2].

#### 3.4.6. Respiratory Depression

Although rare, respiratory depression is one of the most serious opioid-related adverse effects, and it can be lethal. The underlying mechanism involves activation of the mu receptor [11,36], the stimulation of which has an inhibitory effect on CNS neurons that generate respiratory rhythms. Tolerance to this effect usually develops in days or weeks. In individuals receiving chronic opioid treatment, respiratory depression is uncommon and is preceded by sedation. The risk of respiratory depression and death is considerably increased with the coadministration of CNS depressants and opioid therapy [40]. Opioid-induced respiratory distress/depression may be treated with noninvasive respiratory support or naloxone [2,15,16]. Co-prescription of naloxone, an opioid receptor antagonist approved by the US FDA as an over-the-counter nasal spray (Narcan, naloxone hydrochloride, 4 mg) on 29 March 2023 [41], is also recommended for administration by caregivers for respiratory depression and sedation [2]. 

#### 3.4.7. Immunologic Effects/Risk for Infection

Central mu, kappa, and delta opioid receptors have roles in modulating the immune response [18]. Individual opioids have varying effects on immune cells, with morphine showing a strong immunosuppressive effect via inhibition of natural killer cell activity, B and T cell proliferation, macrophage differentiation and function, and proinflammatory cytokine expression in preclinical studies [42]. Opioid administration has known inhibitory effects on antibody and cellular immune responses to increase susceptibility to infection, which may be particularly problematic in the fragile palliative care population. Studies have found a higher risk of invasive pneumococcal diseases with opioid use and a higher incidence of serious infections with long-acting opioids, particularly during the first weeks after treatment initiation [42]. A systematic review and meta-analysis found that prescribed opioid receipt was a risk factor for community-acquired pneumonia in patients with chronic medical conditions and in postoperative patients [43]. Opioids have also been implicated in the increased risk of infections in heroin users and in the pathogenesis of human immunodeficiency virus infection [18]. 

#### 3.4.8. Cardiac Effects

Opioid-induced cardiac effects are not common, though they may be seen more frequently in critically ill patients, and they may occur with supratherapeutic dosing (e.g., overdose) [44]. The opioid-induced cardiac effects of histamine-mediated vasodilation and hypotension are reversible after the administration of naloxone [18]. Methadone is known to prolong the QT interval with standard dosing [18,44] through its inhibition of the cardiac potassium ion channel, KCNH2, to block the rapid delayed rectifier potassium current [45]. The management of patients initiating methadone involves careful monitoring and adherence to expert guidelines to reduce the risk of torsade de pointes [17,45].

## 4. Conclusions

Given that prescription opioids are commonly utilized therapies in the palliative care population, clinician awareness of the risks and potential adverse effects related to their use is tantamount to appropriate pain management in these patients. Common adverse effects of opioids, such as cognitive effects, endocrine and immunologic effects, nausea and vomiting, and sedation, are treated with general approaches such as dose reduction, opioid rotation, changing the route of opioid administration, and symptomatic management [2,17,18]. In contrast, constipation, an opioid-induced adverse effect with a significant incidence (40–80%) that does not have dose-dependent variability [16], is the only opioid-related adverse effect for which there are FDA-approved treatments. PAMORAs were designed specifically to block the pathogenic mechanism of OIC. Currently, methylnaltrexone is the only FDA-approved PAMORA for OIC in the palliative care population. Other opioid-induced side effects that occur less frequently, such as respiratory depression, may have serious and sometimes fatal outcomes if not monitored and addressed promptly [2,15,16]. It is important to emphasize that in the palliative care population, multiple, often overlapping adverse effects could be occurring simultaneously, and patient management will require individualization and diligent monitoring.

For safe prescribing of opioids in the palliative care population, education of patients and caregivers about the risks and potential side effects of opioid therapy is the hallmark of end-of-life care. Continuous monitoring for adverse opioid effects and prompt intervention with appropriate therapies will improve care in this patient population.

## Figures and Tables

**Table 1:** Reported Frequencies of Adverse Effects of Prescription Opioid Treatment [16,18,19,20,21,22,23].

Adverse Effect	Reported Frequency
Cognitive effects	
Memory deficits	73–81%
Sleep disturbance	35–57%
Delirium	21%
Sedation	20–60%
Fatigue	9.5%
Somnolence	7–13%
Endocrine effects	
Hypogonadism (males)	63–69%
Hypocortisolism	15–24%
Gastrointestinal effects	
Constipation	40–80% *
Nausea and vomiting	25–30%
Tolerance (with parenteral opioids)	15%
Myoclonus	3–87%
Pruritus	2–10%
Respiratory depression	
Extradural	0.09–0.2% *
Intrathecal	0.36%
IV PCA	0.2–0.5%
Urinary retention	3.8–18.1%

* Among patients with cancer pain. IV PCA, intravenous patient-controlled analgesia. Adverse effects with unknown frequency include psychomotor, immunologic, and cardiac effects.

**Table 2:** PAMORAs Approved for the Treatment of OIC.

	Methylnaltrexone [31]	Naloxegol [32]	Naldemedine [33]
Indication	Treatment of OIC in adults with advanced illness or pain caused by active cancer who require opioid dosage escalation for palliative care Treatment of OIC in adults with chronic non-cancer pain, including patients with chronic pain related to prior cancer or its treatment who do not require frequent opioid dosage escalation	Treatment of OIC in adult patients with chronic non-cancer pain, including patients with chronic pain related to prior cancer or its treatment who do not require frequent opioid dosage escalation	Treatment of OIC in adult patients with chronic non-cancer pain, including patients with chronic pain related to prior cancer or its treatment who do not require frequent opioid dosage escalation
Route of administration	Oral (chronic non-cancer pain indication in adults) SC (chronic non-cancer pain and advanced illness in adults)	Oral	Oral
Dosing	Adults with chronic non-cancer pain: 450 mg (3, 150-mg tablets) orally once daily in the morning or 12 mg SC once daily Adults with advanced illness: 0.15 mg/kg (<38 or >114 kg), 8 mg (38 to <62 kg), 12 mg (62 to 114 kg)	25 mg (2, 12.5-mg tablets or 1, 25-mg tablet) orally once daily in the morning	0.2 mg (1, 0.2-mg tablet) orally once daily

OIC, opioid-induced constipation; SC, subcutaneous [30,31,32].
